# The Physique of School Teachers

**Published:** 1921-01-01

**Authors:** 


THE PUBLIC WELL-BEING—(*continued*).

### The Physique of School Teachers.

Some interesting points are quoted in *The Times Educational Supplement* from the published results of some analyses carried out in America with regard to the health of students preparing themselves for the teaching profession. The results would have had more value if the investigators, who conducted their inquiries in one school of good standing, had been able to secure actual examinations instead of proceeding by question and answer—a not too reliable method. Still, the subject of the mental and physical health of those who enter the teaching profession is at present one of casual or haphazard consideration, and any attempts to regard the question systematically are to be welcomed. It appears from the questions put to these students in a school in Northern Illinois that some 31 per cent. were not sufficiently vigorous to meet successfully the continuous strain of teaching, while 10 per cent. reported chronic ill-health. Upwards of 30 per cent. reported a decline in health since teaching work was begun, and 79 per cent. reported health disorders within the previous five-year period. A considerable number had become "more nervous." In general the health of the women was found to be poorer than that of the men. Reports from supervisors of these teachers showed that more than a third of them were either irritable, nervous, low in vitality, or affected with other handicaps. One authority went so far as to state his belief that the teaching profession attracted unfit people, and that the teachers' lives developed their unfitness. Especially was this said to be true in regard to nervous diseases, and notably neurasthenia.

Subsequently more careful examinations were carried out with a view to noting defects of so serious a character as actually to interfere with the performance of school life. The examinations were carried out by a physician and a dentist. The following list gives the results of the second series: Deranged digestive organs, 28 per cent.; spine, 9.5; glands, 44; lungs, etc., 1.9; teeth, 40; heart, 1.9; eyes, 28; ears, 8; arms and legs, 38. A surprisingly large number of ear and eye defects were found, and in many instances they were so extreme as to interfere seriously with school work. Yet the pupils were attempting to carry on their duties, often because they could not afford to obtain medical advice and treatment. There was also a notable number of cases of flat foot, some of them due to high heels. "The most discouraging part of the situation," says the writer, "lay in the fact that those young women cared less about their feet than they did about being in style. This is an unfortunate commentary upon the mental set as well as upon the influence of previous years of school and home life in determining ideals of health and physical perfection."

There is here a suggestion that the time may come when intending teachers will be examined not only on the physical side, but as to their temperamental fitness for entering upon a career in which qualities of mind and temper and general habits are just as important as educational qualifications. The conclusion is drawn from these experiments that teachers should be medically examined and treated as systematically as school-children, and that the hygienic environment of schools should come under very strict inspection. There is no doubt that much of the ill-health of teachers occurs after they have actively taken up work of their profession, and that it is due in a large measure to the sedentary nature of their occupation, and to the stuffy conditions in which they have to work. A significant feature of the analyses above recorded is the very low percentage of lung trouble found among students at the age (between eighteen and twenty-one years) when the expectancy is for tuberculosis. It would be valuable to discover whether the disease is markedly more prevalent among teachers after a few years of regular service.

